# Supplementary material for: Acid-treated Staphylococcus aureus induces acute silkworm hemolymph melanization
Source: PLoS One. 2024 May 30;19(5):e0298502. doi: 10.1371/journal.pone.0298502 (PMC11139275; doi:10.1371/journal.pone.0298502)
Supplement: S1 File — (DOCX) [file pone.0298502.s001.docx]

**Acid-treated *Staphylococcus aureus* induces acute silkworm hemolymph melanization**

**Yasuhiko Matsumoto^1*^, Eri Sato^1^, and Takashi Sugita^1^**

^1^Department of Microbiology, Meiji Pharmaceutical University, 2-522-1, Noshio, Kiyose, Tokyo 204-8588, Japan.

*Corresponding author

E-mail: ymatsumoto@my-pharm.ac.jp (YM)

**Short title**: Induction of acute innate immunity in silkworms by acid-treated *Staphylococcus aureus*.


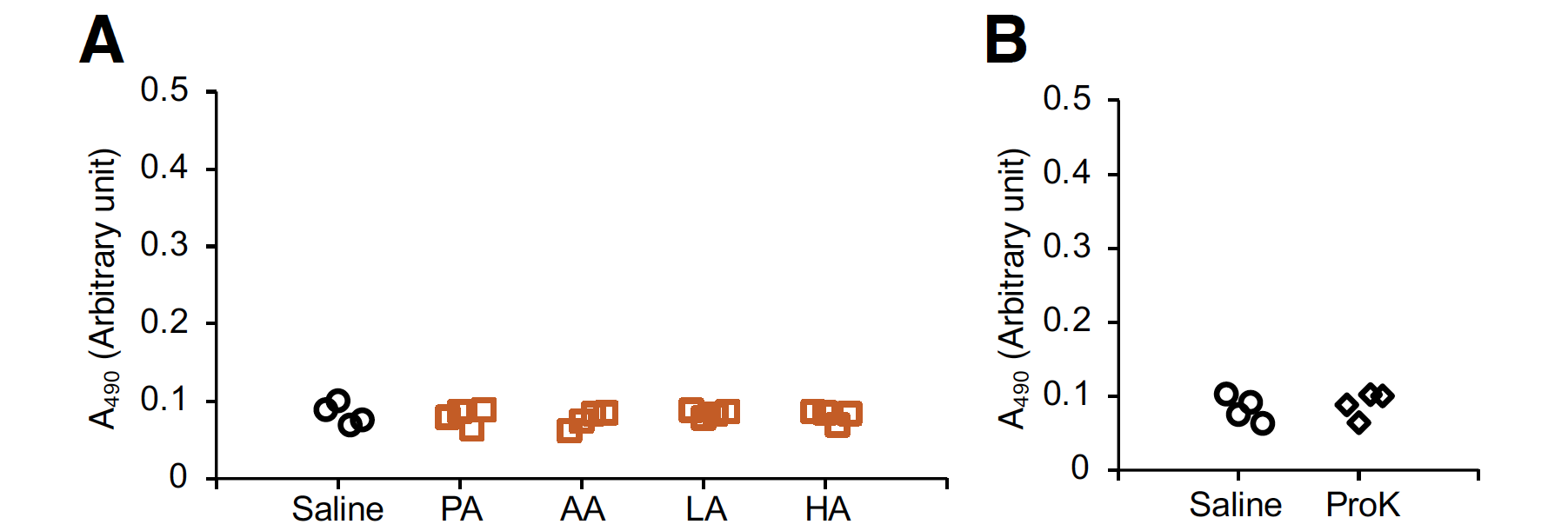


**Supplementary Fig. S1 Effects of short-chain fatty acids and protease K on silkworm hemolymph melanization.**

(**A**, **B**) Sample solution was injected to silkworms and hemolymph was collected at 3 hours after injection. Absorbance of hemolymph samples was measured at 490 nm. (**A**) Samples were saline (Saline), propionic acid (PA) (130 mM), acetic acid (AA) (130 mM), lactic acid (LA) (130 mM), or hydrochloric acid (HA) (130 mM). (**B**) Samples were saline (Saline) or protease K solution (ProK) (0.75 AU/ml). n = 4/group.
