# Supplementary material for: Acid-treated Staphylococcus aureus induces acute silkworm hemolymph melanization
Source: PLoS One. 2024 May 30;19(5):e0298502. doi: 10.1371/journal.pone.0298502 (PMC11139275; doi:10.1371/journal.pone.0298502)
Supplement: Attachment — Submitted filename: Response to reviewers.doc [file pone.0298502.s003.doc]

**Additional Editor Comments:**

1. Fig. 1: Absorbance (at) 490 nm; ℃ is not in correct font style.

According to the editor’s comment, these font styles were revised (Fig. 1).

2. Fig. 2: legend for 2B is incorrect.

According to the editor’s comment, the Figure legend of Fig. 2 was revised (Page 8, lines 146-147).

3. Figs.2-7: Decrease the size of symbols a bit used for data point.

According to the editor’s comment, the size of symbols in Fig. 2-7 was decreased.

4. Method section must be carefully checked to ensure the reproducibility.

According to the editor’s comment, we added the sentence in the Method section of the revised manuscript (Page 6, line 106).

[Page 6, line 106]

Each experiment was performed at least twice to check reproducibility.

5. Please explain the methods for statistical analyses and reasons

According to the editor’s comment, we described the reasons to use these statistical analyses in the Method section of the revised manuscript (Page 6, lines 119-121).

[Page 6, lines 119-121]

The Student's *t*-test was used to assess whether the two groups were statistically significantly different. The Tukey’s test and the Tukey-Kramer test were used to assess whether the multiple groups were statistically significantly different.

6. Please discuss more on the mechanism based on the findings of this study.

Following the editor’s comment, we added the sentences in the Discussion section of the revised manuscript (Page 12, lines 245-250).

[Page 12, lines 245-250]

Proteins of *S. aureus* may be involved in the immune activity induced by acid-treated *S. aureus* in the silkworm evaluation system. The conformational changes of proteins responsible for inducing innate immunity by acid treatment, however, remain unknown. We assumed that *S. aureus* lipoproteins treated with acids lead to conformational changes, and that acid-treated lipoproteins are easily recognized by Toll receptors, which are involved in innate immunity in silkworms. Identification of the responsible proteins and the effects of acid treatment are important issues for future research.

7. The limitations of this study should also be clearly indicated in an additional section.

According to the editor’s comment, we described the limitations in the new paragraph in the Discussion section of the revised manuscript (Page 12, lines 252-256).

[Page 12, lines 251-255]

As a limitation of this study, the effects of acid-treated *S. aureus* on inflammation in individual mammals were not evaluated. It is also unclear whether the effects of an acidic pH on the skin microbiome are related to immune induction. Furthermore, the responsible proteins of *S. aureus* have not yet been identified. The effects of acid treatment and the identification of the responsible proteins are important issues for future research.

Reviewer #1: Silkworms are a suitable model for evaluating the infection and pathogenic mechanism of pathogenic microorganisms. In the manuscript, Matsumoto et al. investigated the hemolymph melanization induced by Staphylococcus aureus in the silkworm model under the participation of Cutibacterium acnes. S. aureus and C. acnes are Gram-positive bacteria present on human skin that can cause inflammatory skin diseases. It is valuable to evaluate the effect of C. acnes on host immunity induced by S. aureus. They found that acidic substances released by C. acnes affect the hemolymph melanization reaction induced by S. aureus. The silkworm hemolymph melanization activity of S. aureus treated with hydrochloric acid was inhibited by protease treatment of S. aureus. I think these results are a beneficial addition to understanding the impact of microbial interactions on the human health of the skin surface. However, there are still some writing logic disorder and incomplete descriptions in this manuscript, some data lack persuasiveness, and the author needs to spend some time carefully revising it.

1.In Figures 4 and 5, the authors should investigate the levels of hemolymph melanization caused by PA, LA, AA, HA, and proteases under the same conditions.

According to the reviewer’s comment, we performed the new experiments (Supplementary Fig. S1). The administration of PA, LA, AA, HA, and proteases did not affect silkworm hemolymph melinization. The Supplementary Fig. 1 was presented in the S1 File. The results were described in the Results section of the revised manuscript (Page 9, lines 174-176, page 11, lines 214-215).

[Page 9, lines 174-176]

On the other hand, administration of these short-chain fatty acid solutions did not affect melanization of the silkworm hemolymph (S1 Fig in S1 File).

[Page 11, lines 214-215]

On the other hand, administration of protease solution did not affect melanization of the silkworm hemolymph (S1 Fig in S1 File).

2.The research results did not show or imply an effect of S. aureus on C. acnes, and the author did not indicate changes in C. acnes during S. aureus-induced folliculitis in the literature. Therefore, further experimental evidence is clearly needed to support the statement that "Preventing the acid-enhanced induction of innate immune activation by S. aureus may contribute to inhibiting the onset of inflammatory skin diseases such as folliculitis."

Following the reviewer’s comment, we added the limitations in this study in the revised manuscript (Page 12, lines 252-256).

[Page 12, lines 252-256]

As a limitation of this study, the effects of acid-treated *S. aureus* on inflammation in individual mammals were not evaluated. It is also unclear whether the effects of an acidic pH on the skin microbiome are related to immune induction. Furthermore, the responsible proteins of *S. aureus* have not yet been identified. The effects of acid treatment and the identification of the responsible proteins are important issues for future research.

3.The introduction is not smoothly written. The third paragraph (lines 60-67) should come after the first paragraph (lines 38-46).

According to the reviewer’s comment, we have changed the paragraph in the revised manuscript (Page 3, lines 47-54).

4.In the Discussion section, there should be further strengthened on how this study deepens our understanding of inflammatory diseases caused by S. aureus. For instance, there exists a vast microbial community on the human skin surface, with S. aureus being an opportunistic pathogen that interacts with other microbes besides C. acnes.

According to the reviewer’s comment, we changed the sentences in the Discussion section of the revised manuscript (Page 12, lines 239-245). The effects of *Delftia acidovorans* was described.

[Page 12, lines 239-245]

On the human skin, various bacteria, including *C. acnes* and *S. aureus*, produce short-chain fatty acids such as lactic acid [32]. Moreover, *S. aureus* and *C. acnes* produce lipases that produce free fatty acids from sebum [13], which can decrease the pH of the skin. On the other hand, bacteria such as *Delftia acidovorans*, a gram-negative bacterium on human skin, produce ammonia that increases the pH [33]. Therefore, the balance of the skin microbiome may affect changes in skin pH. Weak acidity caused by alterations in the skin microbiome may induce an innate immune response by *S. aureus*.

5.The statement "Preventing the acid-enhanced induction of innate immune activation by S. aureus may contribute to inhibiting the onset of inflammatory skin diseases such as folliculitis" in the conclusion should be placed in the discussion."

Following the reviewer’s comment, we moved the sentence in the Discussion section of the revised manuscript (Page 12, lines 250-251).

6.The authors need to provide the silkworm strains used for this study in the Materials and Methods section.

Following the reviewer’s comment, we added the information of the silkworm strain in the Method section in the revised manuscript (Page 5, lines 91-92).

[Page 5, lines 91-92]

Silkworm eggs (Hu Yo × Tukuba Ne) were purchased from Ehime-Sanshu Co. Ltd. (Ehime, Japan), disinfected, and hatched at 25℃ –27℃.

7.Spelling issue. For example, the symbol "℃" in the caption of Figure 1.

According to the reviewer’s comment, the symbol in the Figure legend of Figure 1 were revised (Page 7, line 138). Moreover, the symbol "℃" in the Figure legends of Figure 3-7 also revised (Page 8, line 160, page 9, line 185, page 10, line 195, line, 203, and page 11, line 223).

8.Italics. Line 118, The "P" should be italicized.

According to the reviewer’s comment, the style of “P” in the Method section were changed to italic (Page 6, line 122).
