# Supplementary material for: Acid-treated Staphylococcus aureus induces acute silkworm hemolymph melanization
Source: PLoS One. 2024 May 30;19(5):e0298502. doi: 10.1371/journal.pone.0298502 (PMC11139275; doi:10.1371/journal.pone.0298502)
Supplement: Attachment — Submitted filename: Response to reviewers.doc [file pone.0298502.s004.doc]

Journal Requirements:

Please review your reference list to ensure that it is complete and correct. If you have cited papers that have been retracted, please include the rationale for doing so in the manuscript text, or remove these references and replace them with relevant current references. Any changes to the reference list should be mentioned in the rebuttal letter that accompanies your revised manuscript. If you need to cite a retracted article, indicate the article’s retracted status in the References list and also include a citation and full reference for the retraction notice.

According to the editor’s comment, we checked again the reference list. The reference list was not changed.

**Additional Editor Comments:**

The reviewer and editor feel that your modifications are satisfactory and they pointed out only one minor change of your manuscript (Line 47). Please revise and make a final version of your manuscript to make sure all parts are updated.

According to the editor’s comment, we changed the words of the revised manuscript (Line 47).

[Line 47]

*Cutibacterium acnes*, a gram-positive bacterium on the human skin,

**Reviewer Comments:**

Reviewer #1: Abbreviations should indicate their full name when first appearing in the main text. Line 47, C. acnes.

According to the editor’s comment, we changed the words of the revised manuscript (Line 47).

[Line 47]

*Cutibacterium acnes*, a gram-positive bacterium on the human skin,
